# Nitrogen and Oxygen Isotope Signatures of Nitrogen Compounds during Anammox in the Laboratory and a Wastewater Treatment Plant

**DOI:** 10.1264/jsme2.ME20031

**Published:** 2020-11-07

**Authors:** Shotoku Kotajima, Keisuke Koba, Daisuke Ikeda, Akihiko Terada, Kazuichi Isaka, Kazuya Nishina, Yuuya Kimura, Akiko Makabe, Midori Yano, Hirotsugu Fujitani, Norisuke Ushiki, Satoshi Tsuneda, Muneoki Yoh

**Affiliations:** 1 Graduate School of Agriculture, Tokyo University of Agriculture and Technology, Tokyo, 1838509, Japan; 2 Center for Ecological Research, Kyoto University, Shiga, 5202113, Japan; 3 Institute of Agriculture, Tokyo University of Agriculture and Technology, Tokyo, 1838509, Japan; 4 Graduate School of Engineering, Tokyo University of Agriculture and Technology, Tokyo, 1848588, Japan; 5 Department of Chemical Engineering, Tokyo University of Agriculture and Technology, Tokyo, 1848588, Japan; 6 Institute of Global Innovation Research, Tokyo University of Agriculture and Technology, Tokyo, 1858538, Japan; 7 Hitachi, Ltd., Chiba, 2710064, Japan; 8 Department of Applied Chemistry, Faculty of Science and Engineering, Toyo University, Saitama, 3508585, Japan; 9 Center for Regional Environmental Research, National Institute of Environmental Sciences, Ibaraki, 3058506, Japan; 10 Project Team for Development of New-generation Research Protocol for Submarine Resources, Japan Agency for Marine-Earth Science and Technology, Kanagawa, 2370061, Japan; 11 Present address: Institute for Extra-cutting-edge Science and Technology Avant-garde Research (X-star), Super-cutting-edge Grand and Advanced Research (SUGAR) Program, Japan Agency for Marine-Earth Science and Technology, Kanagawa, 2370061, Japan; 12 Department of Life Science and Medical Bioscience, Waseda University, Tokyo, 1628480, Japan; 13 Present address: Department of Biological Sciences, Faculty of Science and Engineering, Chuo University, Tokyo, 112–8551, Japan

**Keywords:** anammox, stable isotope, nitrite oxidation, isotopic fractionation, denitrification

## Abstract

Isotopic fractionation factors against ^15^N and ^18^O during anammox (anaerobic ammonia oxidization by nitrite) are critical for evaluating the importance of this process in natural environments. We performed batch incubation experiments with an anammox-dominated biomass to investigate nitrogen (N) and oxygen (O) isotopic fractionation factors during anammox and also examined apparent isotope fractionation factors during anammox in an actual wastewater treatment plant. We conducted one incubation experiment with high *δ*^18^O of water to investigate the effects of water *δ*^18^O. The N isotopic fractionation factors estimated from incubation experiments and the wastewater treatment plant were similar to previous values. We also found that the N isotopic effect (^15^ε_NXR_ of –77.8 to –65.9‰ and ^15^Δ_NXR_ of –31.3 to –30.4‰) and possibly O isotopic effect (^18^ε_NXR_ of –20.6‰) for anaerobic nitrite oxidation to nitrate were inverse. We applied the estimated isotopic fractionation factors to the ordinary differential equation model to clarify whether anammox induces deviations in the *δ*^18^O vs *δ*^15^N of nitrate from a linear trajectory of 1, similar to heterotrophic denitrification. Although this deviation has been attributed to nitrite oxidation, the O isotopic fractionation factor for anammox is crucial for obtaining a more detailed understanding of the mechanisms controlling this deviation. In our model, anammox induced the trajectory of the *δ*^18^O vs *δ*^15^N of nitrate during denitrification to less than one, which strongly indicates that this deviation is evidence of nitrite oxidation by anammox under denitrifying conditions.

Anammox (anaerobic ammonia oxidization by nitrite) has been intensively investigated since the discovery of its importance as a N removal process in natural ecosystems (Dalsgaard *et al.*, 2003; Kuypers *et al.*, 2003). The rates of anammox and denitrification are frequently similar (Kuypers *et al.*, 2003; Hamersley *et al.*, 2007; Lam *et al.*, 2009). The detection of anammox in ecosystems is key for further investigations on the relative (quantitative) importance of anammox and denitrification to N losses. Molecular techniques, such as qPCR (Hamasaki *et al.*, 2018) and biomarker analyses (ladderane lipids; Jaeschke *et al.*, 2007), have generally been applied to detect anammox bacteria, followed by ^15^N tracer experiments (Amano *et al.*, 2007) to assess anammox activities (rates) in the laboratory. Although this approach is promising, it only estimates potential anammox rates. Thus, it is crucial to develop screening techniques that estimate anammox in the field.

The naturally occurring stable isotope ratios of N (^15^N / ^14^N, expressed as *δ*^15^N) and O (^18^O / ^16^O, expressed as *δ*^18^O) are useful tracers for investigating the origins, transport, and biogeochemical processes of dissolved inorganic N (DIN), such as nitrate (NO_3_^–^), nitrite (NO_2_^–^), and ammonium (NH_4_^+^), in ecosystems (Casciotti, 2016a, 2016b; Denk *et al.*, 2017; Thuan *et al.*, 2018). Regarding the use of *δ*^15^N and *δ*^18^O to interpret the complex dynamics of DIN, it is essential to apply the isotopic fractionation factors of specific reactions of DIN production and consumption. Previous studies on heterotrophic denitrification estimated ^15^N (Blackmer and Bremner, 1977; Chien *et al.*, 1977; Mariotti *et al.*, 1981, 1982; Bryan *et al.*, 1983; Kawanishi *et al.*, 1993; Barford *et al.*, 2017) and ^18^O fractionation factors (Böttcher *et al.*, 1990; Granger *et al.*, 2008; Kritee *et al.*, 2012; Frey *et al.*, 2014; Martin and Casciotti, 2016; Osaka *et al.*, 2018; Wang *et al.*, 2018). Detailed information on isotopic fractionation during denitrification has encouraged the use of the *δ*^15^N and *δ*^18^O of NO_3_^–^ in investigations on the occurrence and magnitude of denitrification in many ecosystems (Mariotti *et al.*, 1988; Koba *et al.*, 1997; Ostrom *et al.*, 2002; Lehmann *et al.*, 2003; Sigman *et al.*, 2003; Houlton *et al.*, 2006; Houlton and Bai, 2009; Miyajima *et al.*, 2009; Fang *et al.*, 2015; Lennon and Houlton, 2017).

In contrast to denitrification, few studies have used the *δ*^15^N and *δ*^18^O of DIN to examine anammox (Prokopenko *et al.*, 2006; Prokopenko *et al.*, 2013; Wenk *et al.*, 2014; Dähnke and Thamdrup, 2016), and only two studies (Brunner *et al.*, 2013; Kobayashi *et al.*, 2019) have reported isotopic fractionation factors for the anammox reaction. These factors must be known in order to estimate the importance of anammox in a studied ecosystem with the *δ*^15^N and *δ*^18^O of DIN. Brunner *et al.* (2013) estimated a large inverse N isotope effect (*i.e.*, the heavier isotope, ^15^N, reacts faster than the lighter isotope, ^14^N) during NO_3_^–^ production (by anaerobic nitrite oxidation) in anammox as well as a large normal (*i.e.*, the lighter ^14^N reacts faster than the heavier ^15^N) isotope effect for ammonium oxidation, which was confirmed in a later study by Kobayashi *et al.* (2019). However, they only reported the combined ^18^O isotope fractionation factors and do not provide the isotope fractionation factors for the NO_2_^–^ oxidation and its relevant oxygen atom incorporation from water, involved in the combined factors.

Studies on ^15^N and ^18^O fractionation factors revealed that NO_3_^–^ consumption (the assimilatory and dissimilatory reduction of NO_3_^–^) generally induced a 1:1 increase in the *δ*^18^O and *δ*^15^N of NO_3_^–^ (Granger *et al.*, 2008; Granger *et al.*, 2010; Karsh *et al.*, 2012; Rohde *et al.*, 2015; Osaka *et al.*, 2018). This finding prompted the use of the *δ*^18^O and *δ*^15^N of NO_3_^–^ to detect NO_3_^–^ consumption in the actual ecosystem as well as investigations on NO_3_^–^ isotope anomalies, specifically isotopic deviations from a slope of 1 in the *δ*^18^O vs *δ*^15^N of NO_3_^–^ (Δ[15, 18]; Sigman *et al.*, 2005), in order to deepen insights into NO_3_^–^ dynamics (Casciotti *et al.*, 2008; Casciotti and Buchwald, 2012; Bourbonnais *et al.*, 2013; Peters *et al.*, 2018; White *et al.*, 2019). Granger and Wankel (2016) proposed that widely observed deviations in the *δ*^18^O vs *δ*^15^N of NO_3_^–^ from the denitrification slope of 1 in freshwater systems (Sigman *et al.*, 2005; Granger *et al.*, 2008; Kritee *et al.*, 2012) must result from concurrent NO_3_^–^ production (nitrification or anammox) in the denitrifying system that has been largely overlooked. However, they lacked information on the ^18^O fractionation factor for anammox, and assumed that the ^18^O fractionation factor during NO_3_^–^ production by anammox was similar to that for aerobic nitrite oxidation to NO_3_^–^ by nitrifiers (nitrification). Thus, it is essential to investigate the ^15^N and ^18^O fractionation factors during anammox not only for the better use of the *δ*^18^O and *δ*^15^N of NO_3_^–^ in anammox studies, but also to obtain a more detailed understanding of ^15^N and ^18^O fractionation.

We herein report unique data on ^18^O fractionation factors during anammox. We calculated apparent ^15^N and ^18^O fractionation factors with data collected from a wastewater treatment plant (WWTP) at which anammox reactors were installed at the final stage of treatment (Isaka *et al.*, 2017). We also performed anaerobic laboratory incubations with an anammox-dominated biomass to obtain more information on isotopic fractionation during anammox. We conducted one incubation experiment with high *δ*^18^O of water to investigate the effects of water *δ*^18^O. We then simulated system behavior with the observed isotopic fractionation factors to establish whether deviations in the *δ*^18^O vs *δ*^15^N of NO_3_^–^ from the denitrification slope of 1 may be used to detect anammox activity.

## Materials and Methods

### Full-scale anammox wastewater treatment plant

Influent and treated water in the full-scale anammox wastewater treatment plant (Isaka *et al.*, 2017) were sampled three times (28th April, 7th and 12th May 2015). Detailed information on water chemistry and plant performance have been reported by Isaka *et al.* (2017). The anammox plant consists of a denitrifier reactor (DN), biochemical oxygen demand (BOD) oxidation reactor (BD), nitrite-nitrification reactor (NT), and anammox reactor (ANX). We collected water samples from each reactor (Fig. S1). The wastewater introduced into this anammox plant was effluent from an ammonia plant, which was pure water containing mainly NH_4_^+^ and methanol. Average NH_4_^+^ and total organic concentrations were 658 and 37‍ ‍mg L^–1^, respectively (Isaka *et al.*, 2017). Solutions were sampled from these reactors (Fig. S1) to measure the concentrations and isotope ratios of DIN. Samples (10‍ ‍mL each) were immediately filtered through 0.45-μm disk filters (25CS045AN; ADVANTEC Toyo Kaisha) and then collected in plastic centrifuge tubes. Samples were frozen until further measurements.

### Biomass incubation experiments

We performed batch incubations with anammox bacteria. Details on a small-scale anammox reactor with activated sludge, including start-up information, maintenance, performance, input solutions, and microbial communities of the reactor, are provided in Supplemental Information (SI Text 1.1).

In the first experiment (Experiment A), the biomass in the reactor was sampled and incubated with the media used for the reactor, while the sampled biomass was re-suspended in fresh, chemically defined media in the second (Experiment B) and third (Experiment C) experiments. Difficulties were associated with performing incubation experiments with the anammox biomass for isotopic measurements, and, thus, we employed slightly different settings and operations to facilitate constant and active anammox reactions. In each experiment, 15‍ ‍mL of the biomass suspension in media solution was filtered with filter paper (Reeve Angel, Whatman) and differences in filter weights before and after filtration were used to calculate the suspended solid (S.S.) concentration after the filter had been oven-dried (at 105°C).

### Experiment A

The biofilm and incubation media solution (500‍ ‍mL in total) were sampled from the incubation membrane in the anammox reactor (SI Text 1.1). The incubation was performed anaerobically in the glovebox at room temperature (25–30°C) after the purging of media by N_2_ gas to remove dissolved oxygen (DO). pH, DO, and the concentrations of NH_4_^+^ and NO_2_^–^ were regularly measured to confirm the anammox activity of the biofilm, and pH (8.0) was maintained by adding KH_2_PO_4_ and Na_2_HPO_4_·12H_2_O solution. After the addition of NaNO_2_, (NH_4_)_2_SO_4_, and NaHCO_3_, we started the incubation and sampled 10‍ ‍mL of media. Sampled media were filtered with a 0.20-μm syringe filter and then split into three; one for NO_3_^–^ followed by the removal of NO_2_^–^ (Granger and Sigman, 2009), another one for NO_2_^–^ with high pH by the addition of 2M NaOH solution to prevent oxygen atom exchange between NO_2_^–^ and water (Bourbonnais *et al.*, 2017), and one for NH_4_^+^ with low pH by the addition of 4.8 M H_2_SO_4_ to prevent NH_4_^+^ from volatilizing. These subsamples were frozen (–30°C) until further analyses.

### Experiment B

The granule biomass that accumulated at the bottom of the anammox reactor was sampled. Granules were rinsed anaerobically with new media (N_2_ purged) in the glovebox. Media consisted of NaHCO_3_ 502‍ ‍mg L^–1^; MgSO_4_·7H_2_O 603‍ ‍mg L^–1^; CaCl_2_ 180.5‍ ‍mg L^–1^; KH_2_PO_4_ 169‍ ‍mg L^–1^; Na_2_HPO_4_·12H_2_O 282‍ ‍mg L^–1^; trace elements of solution I (containing EDTA 6.369‍ ‍g L^–1^; FeSO_4_·7H_2_O 9.14‍ ‍g L^–1^) 0.5‍ ‍mL and solution II (containing EDTA 19.106‍ ‍g L^–1^; ZnSO_4_·7H_2_O 0.43‍ ‍g L^–1^; CoCl_2_·6H_2_O 0.24‍ ‍g L^–1^; MnCl_2_·4H_2_O 0.99‍ ‍g L^–1^; CuSO_4_·5H_2_O 0.25‍ ‍g L^–1^; Na_2_MoO_4_·2H_2_O 0.22‍ ‍g L^–1^; NiCl_2_·6H_2_O 0.19‍ ‍g L^–1^; Na_2_SeO_4_·10H_2_O 0.21‍ ‍g L^–1^; H_3_BO_3_ 0.014‍ ‍g L^–1^) 0.5‍ ‍mL. We added NaNO_2_ and (NH_4_)_2_SO_4_ to media (500‍ ‍mL) with the anammox granules and then started the incubation at room temperature (25–30°C). We monitored pH (7.9 to 8.8) and NO_2_^–^ to assess the progress of anammox. Sampling was performed as described in Experiment A.

### Experiment C

We incubated the biofilm collected from the incubation membrane in the anammox reactor with the same media used in Experiment B; however, the *δ*^18^O of water (*δ*^18^O_H2O_) was markedly higher (229‰) than that in Experiments A and B (–8‰). This “heavy” water was prepared by mixing ^18^O-labeled water (10% atom ^18^O) with Milli-Q water. During the incubation, media with biofilms were shaken at a constant temperature (30°C) and continuously purged with a gas mixture (95% Ar + 5% CO_2_) to maintain low DO levels. pH ranged between 7.1 and 7.5 and the monitoring and sampling scheme was identical to Experiment B

### Chemical analysis

DIN concentrations in water samples from the anammox plant and incubation experiments were measured using colorimetric methods with an autoanalyzer (Quatro, BL-Tec) (Thuan *et al.*, 2018) after appropriate dilutions. In Experiment A, NH_4_^+^ concentrations were measured during the incubation by the o-phthaldialdehyde (OPA) method (Holmes *et al.*, 1999). The DO and pH of the incubation media were monitored during the incubation with a DO meter (HQ30d; Hach) and pH meter (D-71; Horiba).

*δ*^15^N and *δ*^18^O values were assessed by GC-IRMS (Sercon 20–22 with Cryoprep) (Thuan *et al.*, 2018) with the denitrifier method (Sigman *et al.*, 2001; Casciotti *et al.*, 2002) for NO_3_^–^ (*δ*^15^N_NO3–_ and *δ*^18^O_NO3–_, respectively) with USGS 32, 34, 35, and IAEA-2 as standards, and with the azide method (McIlvin and Altabet, 2005) for NO_2_^–^ (*δ*^15^N_NO2–_ and *δ*^18^O_NO2–_, respectively) with TUAT-NO2-1 to TUAT-NO2-5 (Thuan *et al.*, 2018) calibrated against N-23, N-7373, and N-10219 (Casciotti *et al.*, 2007) as the standards. Analytical precision (expressed as the standard deviation of repeatedly measured samples) was ±0.2‰ for *δ*^15^N_NO2–_ and *δ*^15^N_NO3–_, and ±0.5‰ for *δ*^18^O_NO2–_ and *δ*^18^O_NO3–_. The *δ*^15^N values of NH_4_^+^ (*δ*^15^N_NH4+_) were evaluated using GC-IRMS with the denitrifier method after the conversion of NH_4_^+^ to NO_3_^–^ by persulfate oxidation (Koba *et al.*, 2012; Thuan *et al.*, 2018) with USGS 25, 26, and IAEA-N-2 as the standards. Analytical precision was ±0.5‰ for the *δ*^15^N of NH_4_^+^. Water with high *δ*^18^O (229‰; measured by GC-IRMS with the modified azide method; McIlvin and Casciotti, 2006; Thuan *et al.*, 2018) from Experiment C was used to prepare NO_3_^–^ and NO_2_^–^ isotope standards for NO_3_^–^ and NO_2_^–^ measurements in order to correct for the effects of oxygen atom incorporation during the analysis. *δ*^15^N and *δ*^18^O are expressed as (R_SampleN/R_Nitrogen)–1 and (R_SampleO/R_Oxygen)–1 where R_SampleN and R_SampleO are [^15^N/^14^N] and [^18^O/^16^O] of the sample, respectively, R_Nitrogen is [^15^N/^14^N] of atmospheric N_2_ and R_Oxygen is [^18^O/^16^O] of Vienna Standard Mean Ocean Water (Table S1).

### Calculation of apparent isotopic fractionation factors for the anammox plant

Apparent isotopic fractionation factors regarding anammox in the anammox plant were calculated as described by Kobayashi *et al.* (2019) based on steady-state, open-system isotope systematics reported by Fry (2006).

### Apparent N isotope effects of the ammonium oxidation to N_2_, and nitrite reduction and oxidation for the anammox plant

The ammonium oxidation to N_2_ by NO_2_^–^ has isotope fractionation defined as ^15^Δ_AMX_. The *δ*^15^N of influx NH_4_^+^ (*δ*^15^N_NH4+_NT_), residual NH_4_^+^ (*δ*^15^N_NH4+_ANX_), and the fraction of NH_4_^+^ reacting (*f_NH4+_*) in ANX reactor (Fig. S1) at a steady state are used to estimate ^15^Δ_AMX_ (Fry, 2006; Kobayashi *et al.*, 2019):

^15^Δ_AMX_ = (*δ*^15^N_NH4+_ANX_ – *δ*^15^N_NH4+_NT_) / *f_NH4+_* --- eq. (1)

*f_NH4+_* = ([NH_4_^+^]_NT_ – [NH_4_^+^]_ANX_) / [NH_4_^+^]_NT_

where [NH_4_^+^]_NT_ and [NH_4_^+^]_ANX_ are the NH_4_^+^ concentrations in NT and ANX reactors, respectively.

The *δ*^15^N of NO_2_^–^, NO_3_^–^, and N_2_ in ANX reactor (*δ*^15^N_NO2–_ANX_, *δ*^15^N_NO3–_ANX_, and *δ*^15^N_N2_ANX_, respectively) at the steady state are given as follows (Fry, 2006; Kobayashi *et al.*, 2019):

^15^Δ_AMXNIR_ = *δ*^15^N_NO2–_ANX_ – *δ*^15^N_N2_ANX_ --- eq. (2)

^15^Δ_NXR_ = *δ*^15^N_NO2–_ANX_ – *δ*^15^N_NO3–_ANX_ --- eq. (3)

where ^15^N fractionation for nitrite reduction in anammox and nitrite oxidation are ^15^Δ_AMXNIR_ and ^15^Δ_NXR_, respectively.

*δ*^15^N_NO2–_NT_ is defined as:

*δ*^15^N_NO2–_NT_ = (1 – *a* – *b*) × *δ*^15^N_N2_ANX_ + *a* × *δ*^15^N_NO2–_ANX_ + *b* × *δ*^15^N_NO3–_ANX_ --- eq. (4)

with

*a* = [NO_2_^–^]_ANX_ / ([NO_2_^ – ^]_NT_ – [NO_2_^–^]_ANX_)

*b* = ([NO_3_^–^]_ANX_ – [NO_3_^–^]_NT_) / ([NO_2_^–^]_NT_ – [NO_2_^–^]_ANX_)

where *δ*^15^N_NO2–_NT_ is *δ*^15^N_NO2–_ in NT reactor and the concentrations of NO_2_^–^ and NO_3_^–^ in NT and ANX reactors are [NO_2_^–^]_NT_, [NO_2_^–^]_ANX_, [NO_3_^–^]_NT_, and [NO_3_^–^]_ANX_, respectively.

The combination of eqs. (2) and (4) gives

*δ*^15^N_NO2–_NT_ = (1 – *a* – *b*) × *δ*^15^N_N2_ANX_ + *a* × *δ*^15^N_NO2–_ANX_ + *b* × *δ*^15^N_NO3–_ANX_

= (1 – *a* – *b*) × (*δ*^15^N_NO2–_ANX_ – ^15^Δ_AMXNIR_) + *a* × *δ*^15^N_NO2–_ANX_ + *b* × *δ*^15^N_NO3–_ANX_

= *δ*^15^N_NO2–_ANX_ – ^15^Δ_AMXNIR_ – *a* × *δ*^15^N_NO2–_ANX_+ *a* × ^15^Δ_AMXNIR_ – *b* × *δ*^15^N_NO2–_ANX_ + *b* × ^15^Δ_AMXNIR_+ *a* × *δ*^15^N_NO2–_ANX_ + *b* × *δ*^15^N_NO3–_ANX_

= *b* × (*δ*^15^N_NO3–_ANX_ – *δ*^15^N_NO2–_ANX_) + *δ*^15^N_NO2–_ANX_+ ^15^Δ_AMXNIR_ × (*a* + *b* – 1)

= – (*b* × ^15^Δ_NXR_) + *δ*^15^N_NO2–_ANX_ + ^15^Δ_AMXNIR_ × (*a* + *b* – 1)

^15^Δ_AMXNIR_ = [*δ*^15^N_NO2–_ANX_ – *δ*^15^N_NO2–_NT_ – *b* × ^15^Δ_NXR_] / (*a* + *b* – 1) --- eq. (5)

### Apparent combined O isotope effect of nitrite oxidation for the anammox plant

To calculate ^18^O fractionation during nitrite oxidation to nitrate, we followed the approach described by Kobayashi *et al.* (2019) to calculate combined isotope fractionation (^18^E_AMXcombined_) because of the lack of detailed information on isotopic fractionation for the nitrite oxidation and oxygen atom incorporation during nitrite oxidation. Thus, we calculated ^18^E_AMXcombined_ as follows:

^18^E_AMXcombined_ = 2/3 *δ*^18^O_NO2–_ANX_ + 1/3 *δ*^18^O_H2O_ – *δ*^18^O_NO3–_ANX_ --- eq. (6)

where *δ*^18^O_NO2–_ANX_, *δ*^18^O_H2O_, and *δ*^18^O_NO3–_ANX_ are the ^18^O ratios of NO_2_^–^, water, and NO_3_^–^ in ANX reactor, respectively.

### Calculation of isotopic fractionation factors for incubations and a simulation with the dynamic model (the anammox model)

**Fig. 1.**
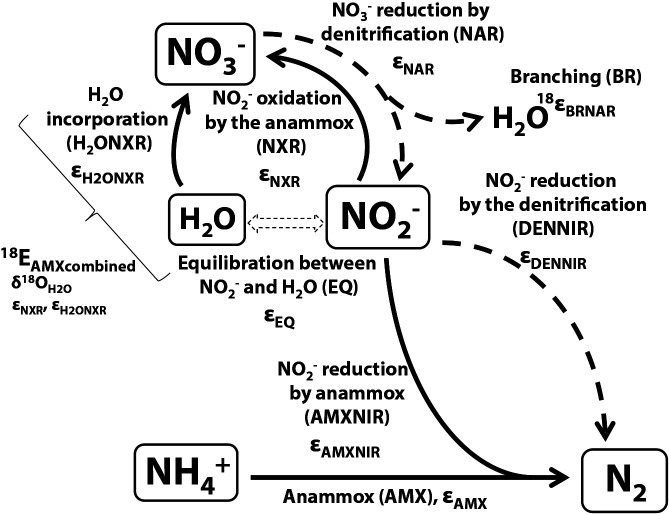
Schematic of the anammox and denitrification system. Dotted arrows indicate denitrification processes that were not included in the anammox model.

We developed an ordinary differential equation model as described by Casciotti and Buchwald (2012), Granger and Wankel (2016), and He and Bao (2019). We prepared the model (the anammox model) to calculate the isotopic fractionation factors for Experiments A, B, and C. The N transformations and associated isotopic fractionation (Fig. 1) were implemented in the anammox model with Berkeley Madonna (BM) software (Macey *et al.*, 2000), with a 4th-order Runge–Kutta method for integration. We initially used the curve-fitting function in BM software (least squares fitting) to calculate the rate constant of the ammonium oxidation based on concentration data in each experiment. Isotopic fractionation factors and the exchange rate of oxygen atoms between water and NO_2_^–^ were then estimated from isotopic data.

Fluxes regarding the anammox process (Fig. 1) are defined as

AMX = AMXNIR = *k*_AMO14N_ × [^14^NH_4_^+^] --- eq. (7)

NXR = AMXNIR × (*x* / (1 – *x*)) --- eq. (8)

where AMX, NXR, and AMXNIR are the (^14^N) fluxes of ammonium oxidation, nitrite oxidation, and reduction by anammox (Fig. 1), *k*_AMO14N_ is the rate constant for AMX, and *x* is a stoichiometric ratio (increase in [NO_3_^–^]/decrease in [NO_2_^–^]) (Brunner *et al.*, 2013). We omitted the two N transformation processes regarding denitrification (nitrate and nitrite reduction by denitrification, NAR, and DENNIR, respectively; Fig. 1) in the anammox model because of the small contributions of denitrifying bacteria to the total microbial community (Fig. S2) and the small contribution of denitrification of only 5–10% at most to the total N removal rate in this study (estimated by ^15^N tracer measurements; D. Ikeda, personal communications).

Regarding NO_2_^–^;

d/dt [^14^NO_2_^–^] = – NXR – AMXNIR --- eq. (9)

d/dt [^15^NO_2_^–^] = – (R_NitriteN × NXR / ^15^ε_NXR_)– (R_NitriteN × AMXNIR / ^15^ε_AMXNIR_) --- eq. (10)

d/dt [N^16^O_2_^–^] = – 2NXR – 2AMXNIR --- eq. (11)

d/dt [N^16^O^18^O^–^] = – (R_NitriteO × 2NXR / ^18^ε_NXR_)– (R_NitriteO × 2AMXNIR / ^18^ε_AMXNIR_)– N^16^O^18^O^–^_exch_OUT_ + N^16^O^18^O^–^_exch_IN_

= – (R_NitriteO × 2NXR / ^18^ε_NXR_)– (R_NitriteO × 2AMXNIR / ^18^ε_AMXNIR_)– *k*_exch_ × [N^16^O^18^O^–^] + *k*_exch_×R_WaterO / ^18^ε_EQ_ --- eq. (12)

where R_NitriteO, R_NitrateO, R_NitriteN, and R_NitrateN are the ^18^O/^16^O and ^15^N/^14^N of [NO_2_^–^] and [NO_3_^–^], respectively. R_WaterO is the [^18^O/^16^O] of H_2_O. ^15^ε_NXR_ and ^15^ε_AMXNIR_ are the ^15^N fractionation factors of NXR and AMXNIR. ^18^ε_NXR_ and ^18^ε_AMXNIR_ are the ^18^O fractionation factors of NXR and AMXNIR, respectively. ^18^ε_EQ_, the ^18^O fractionation factor of the equilibration between NO_2_^–^ and H_2_O, was set at 13‰ in the present study based on the incubation temperature and pH (Table S1; Buchwald and Casciotti, 2013). We applied *k*_exch_ (rate coefficient for oxygen atom exchange), N^16^O^18^O^–^_exch_OUT_, and N^16^O^18^O^–^_exch_IN_ (N^16^O^18^O^–^ efflux and influx regarding the N^16^O^18^O^–^ pool, respectively) as described by He and Bao (2019) to implement oxygen atom exchange rates between NO_2_^–^ and H_2_O.

Regarding NO_3_^–^;

d/dt [^14^NO_3_^–^] = NXR --- eq. (13)

d/dt [^15^NO_3_^–^] = (R_NitriteN × NXR / ^15^ε_NXR_) --- eq. (14)

d/dt [N^16^O_3_^–^] = 3 NXR --- eq. (15)

d/dt [N^18^O^16^O_2_^–^] = (R_NitriteO × 2 NXR / ^18^ε_NXR_)+ (R_WaterO × NXR) / ^18^ε_H2ONXR_) --- eq. (16)

where ^18^ε_H2ONXR_ (assigned as 10.0‰; Table S1; Buchwald and Casciotti, 2010; Casciotti and Buchwald, 2012) is the ^18^O fractionation factor for the incorporation of oxygen from H_2_O into NO_3_^–^ during the NXR reaction (Fig. 1).

Regarding NH_4_^+^;

d/dt [^14^NH_4_^+^] = – AMX --- eq. (17)

d/dt [^15^NH_4_^+^] = – (R_AmmoniumN × AMX/ ^15^ε_AMX_) --- eq. (18)

where R_AmmoniumN is the ^15^N / ^14^N of [NH_4_^+^] and ^15^ε_AMX_ is the N isotopic fractionation factor for NH_4_^+^ consumption by anammox (Fig. 1).

The approximate stoichiometry of the anammox process converting NO_2_^–^ and NH_4_^+^ to N_2_ and NO_3_^–^ is as follows (Brunner *et al.*, 2013):

1.3NO_2_^–^ + 1NH_4_^+^ → 1N_2_ + 0.3NO_3_^–^ + 2H_2_O --- eq. (19)

However, this stoichiometry between nitrite removal and nitrate production has been reported to vary (Brunner *et al.*, 2013). Thus, we estimated this stoichiometry (*x*) together with *k*_AMO14N_ with concentration data, which provided the AMX, AMXNIR, and NXR fluxes used in the calculation above (eq. [7] and [8]; Table S1). After estimating *x* and *k*_AMO14N_, we estimated the *k*_exch_ of oxygen atoms between H_2_O and NO_2_^–^ (Table S1) using the curve-fitting functions for Experiments A and B. In Experiment C with high *δ*^18^O_H2O_, we performed another incubation without the anammox biofilm (Fig. S3) to measure *k*_exch_. At the same time, we estimated other isotopic fractionation factors (^15^ε_AMXNIR_, ^15^ε_NXR_, ^15^ε_AMX_, ^18^ε_AMXNIR_, and ^18^ε_NXR_). We assigned the range from 0 to 60‰ (with 5 and 10‰ as the initial values for the curve-fitting function of BM software) to estimate isotopic fractionation factors. We considered this 60‰ range for the curve-fitting estimate to be reasonable because isotopic fractionation factors larger than 60‰ are rarely observed (Denk *et al.*, 2017). It is important to note that curve-fitting for Experiments B and C was not successfully achieved for ^18^ε_AMXNIR_, resulting in extremely high or low estimated values (calculated ^18^ε_AMXNIR_ values were 0 and 60‰ for Experiments B and C, respectively. In addition, ^18^ε_NXR_ (calculated as –11.2 and –84.3‰ for Experiments B and C, respectively) and consequently ^18^E_AMXcombined_ (calculated as –4.2 and –52.9‰ for Experiments B and C, respectively), were not all successfully estimated for Experiments B and C. Based on these uncertainties in parameter estimations, we did not report these calculated values for Experiments B and C; however, we speculate that these calculated parameter sets support ^18^ε_AMXNIR_ as normal and ^18^ε_NXR_ being inverse isotope fractionation, as discussed below for Experiment A. The curve-fitting function (“multiple-fit” in BM software) (Macey *et al.*, 2000) was applied with a tolerance of 1 × 10^–6^. BM codes for the anammox model for curve fittings with concentrations and isotopic data are provided in the Zenodo website (https://doi.org/10.5281/zenodo.3895346) and Table S2 showed the root mean square errors (RMSE) for concentrations and isotope values for the fitted model.

### Simulation exercise for denitrification and anammox (the anammox-denitrification model)

**Fig. 3.**
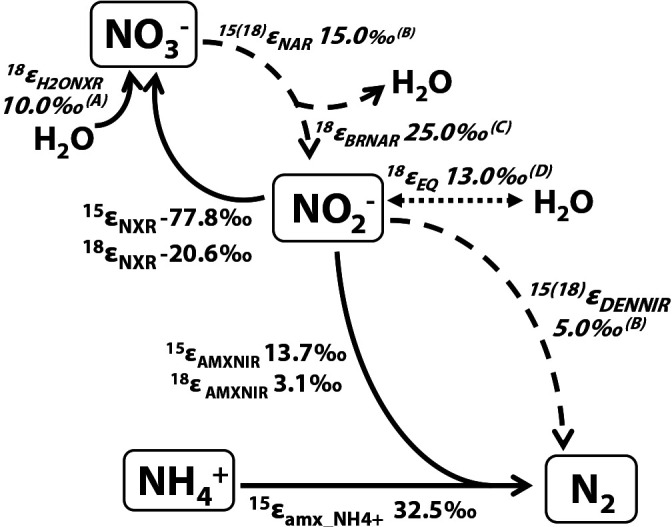
Isotopic fractionation factors applied in the anammox-denitrification simulation model. These factors were from Experiment A or previous studies; (A) Buchwald and Casciotti, 2010; (B) Granger and Wankel, 2016; (C) Casciotti *et al.*, 2002; (D) Buchwald and Casciotti, 2013.

We added the fluxes of denitrification (NAR and DENNIR; Fig. 3) to the anammox model in order for the anammox-denitrification model to simulate anammox and denitrification as follows:

Regarding NO_2_^–^;

d/dt [^14^NO_2_^–^] = – NXR + NAR – DENNIR – AMXNIR --- eq. (20)

d/dt [^15^NO_2_^–^] = – (R_NitriteN × NXR / ^15^ε_NXR_)+ (R_NitrateN × NAR / ^1518^ε_NAR_)– (R_NitriteN × DENNIR / ^15^ε_DENNIR_)– (R_NitriteN × AMXNIR / ^15^ε_AMXNIR_) --- eq. (21)

d/dt [N^16^O_2_^–^] = – 2 NXR + 2 NAR – 2 DENNIR – 2 AMXNIR --- eq. (22)

where ^1518^ε_NAR_ (assigned as 15‰, Granger *et al.*, 2008; Table S1) is the N and O isotopic fractionation factor of NAR (*i.e.*, ^15^ε_NAR_ = ^18^ε_NAR_, Sigman *et al.*, 2005; Granger *et al.*, 2008; Granger *et al.*, 2010; Rohde *et al.*, 2015; Osaka *et al.*, 2018) and ^15^ε_DENNIR_ (assigned as 5‰, Granger and Wankel, 2016; Table S1) is the ^15^N fractionation factor of DENNIR.

In the case of no exchange of oxygen atoms between NO_2_^–^ and H_2_O,

d/dt [N^16^O^18^O^–^] = – (R_NitriteO × 2 NXR / ^18^ε_NXR_)+ (R_NitrateO × 2 NAR / ^1518^ε_NAR_) / ^18^ε_H2OBRNAR_– (R_NitriteO × 2 DENNIR / ^18^ε_DENNIR_)– (R_NitriteO × 2 AMXNIR / ^18^ε_AMXNIR_) --- eq. (23a)

where ^18^ε_H2OBRNAR_ is the ^18^O fractionation factor for the “branching effect” (assigned as 25‰, Casciotti and McIlvin, 2007; Table S1) during NAR (Fig. 1).

In the case of full exchange between NO_2_^–^ and H_2_O,

d/dt [N^16^O^18^O^–^] = d/dt [N^16^O_2_^–^] × R_Oxygen × [(*δ*^18^O_NO2–_EQ_ / 1000) + 1] --- eq. (23b)

where *δ*^18^O_NO2–_EQ_ is *δ*^18^O_NO2–_ at the equilibrium with H_2_O (= *δ*^18^O_H2O_ + ^18^ε_EQ_) and *δ*^18^O_NO2–_ is always set to *δ*^18^O_NO2–_EQ_.

Regarding NO_3_^–^;

d/dt [^14^NO_3_^–^] = NXR – NAR --- eq. (24)

d/dt [^15^NO_3_^–^] = (R_NitriteN × NXR / ^15^ε_NXR_) – (R_NitrateN × NAR / ^1518^ε_NAR_) --- eq. (25)

d/dt [N^18^O^16^O_2_^–^] = 3 NXR – 3 NAR --- eq. (26)

d/dt [N^18^O^16^O_2_^–^] = (R_NitriteO × 2 NXR / ^18^ε_NXR_)+ (R_WaterO × NXR / ^18^ε_H2ONXR_)– (R_NitrateO × 3 NAR / ^1518^ε_NAR_) --- eq. (27)

We applied the estimated isotopic fractionation factors from Experiment A (Table 2) together with the reported values for fractionation factors (Fig. 3) to simulate whether the stronger contribution of anammox to denitrification alters the slope of the *δ*^18^O vs *δ*^15^N of NO_3_^–^ from the denitrification slope of 1 with or without oxygen atom exchange between H_2_O and NO_2_^–^ in freshwater (*δ*^18^O_H2O_ = –8‰) or seawater (0‰) environments. The BM code for the anammox and anammox-denitrification models is provided on the Zenodo website (https://doi.org/10.5281/zenodo.3895346).

## Results and Discussion

### Anammox plant data

Throughout the 17-day span of the three sampling times, the DIN concentrations and their isotopic signatures were stable (Table 1) for each reactor. Stoichiometries for the anammox process were calculated by changes in DIN concentrations between NT and ANX reactors (decreases in the concentrations of NO_2_^–^ and NH_4_^+^ ΔNO_2_^–^ / ΔNH_4_^+^, for NO_2_^–^ consumption, and an increase in NO_3_^–^ with a decrease in NO_2_^–^, ΔNO_3_^–^ / ΔNH_4_^+^, for NO_3_^–^ production). ΔNO_2_^–^ / ΔNH_4_^+^ ranged 1.22 ~ 1.26 and ΔNO_3_^–^ / ΔNH_4_^+^ was 0.14 ~ 0.15, both of which were within the range for anammox reactions (1.03 to 1.32 for ΔNO_2_^–^ / ΔNH_4_^+^ and 0.14 to 0.35 for ΔNO_3_^–^ / ΔNH_4_^+^) (Yao *et al.*, 2015). Isaka *et al.* (2017) also reported that ΔNO_2_^–^ / ΔNH_4_^+^ was 1.23 from this anammox plant, which indicates the appropriate performance of anammox in ANX reactor. The ammonium in the influent with a high concentration (44.5‍ ‍mM) and low *δ*^15^N_NH4+_ (–10.4‰) was gradually consumed in the reactors with normal isotopic fractionation (*i.e.*, with increasing *δ*^15^N), resulting in a low concentration (1.9‍ ‍mM) with high *δ*^15^N_NH4+_ (50.2‰) at final ANX reactor (Table 1). Nitrite produced in DN, BD, and NT reactors and consumed in ANX reactor had low *δ*^15^N_NO2–_ values (–38.8 to –21.6‰) and relatively stable *δ*^18^O_NO2–_ values (3.3 to 4.7‰; Table 1). Nitrate was not produced before ANX reactor and *δ*^15^N_NO3–_ (9.3‰) was higher than *δ*^15^N_NO2–_ (–21.6‰), with no significant difference between *δ*^18^O_NO3–_ and *δ*^18^O_NO2–_ in ANX (Table 1). In comparisons with the isotopic data for other types of WWTP (Table 1), we found that the lower *δ*^18^O_NO3–_ and higher *δ*^15^N_NH4+_ from the anammox plant was useful for tracking the fate of N derived from the anammox wastewater plant.

The calculated ^15^Δ_AMX_ was large (34.0 ~ 34.8‰; Table 2), which was similar to the reported value for anammox (30.9 ~ 32.7‰; Kobayashi *et al.*, 2019) and to the isotope effect for aerobic ammonia oxidization (29.6 ± 4.9‰; Denk *et al.*, 2017). The two NO_2_^–^ consumption pathways in the ANX reactor had different ^15^N fractionation; normal (positive), large ^15^Δ_AMXNIR_ (11.8 ~ 12.4‰), and inverse (negative) ^15^Δ_NXR_ (–30.4 ~ –31.3‰), which fell within reported values (Kobayashi *et al.*, 2019) (Table 2). The present results confirmed an inverse ^15^N effect during anaerobic NO_2_^–^ oxidation to NO_3_^–^, as previously reported (Brunner *et al.*, 2013; Kobayashi *et al.*, 2019) for aerobic NO_2_^–^ oxidation to NO_3_^–^ (Casciotti, 2009; Buchwald and Casciotti, 2010). Similarly, the small and negative apparent “combined” ^18^O fractionation for ammonium oxidization by NO_2_^–^ (–2.5 ~ –3.8‰; ^18^E_AMXcombined_) also fell within the reported range of –1.5 to –‍12‰ (Kobayashi *et al.*, 2019) (Table 2). The negative ^18^E_AMXcombined_ values reported here and by Kobayashi *et al.* (2019) during NO_3_^–^ production in anammox agree with the inverse ^18^O fractionation for aerobic nitrite oxidation to NO_3_^–^ (Casciotti, 2009; Buchwald and Casciotti, 2010).

### Incubation experiments

**Fig. 2.**
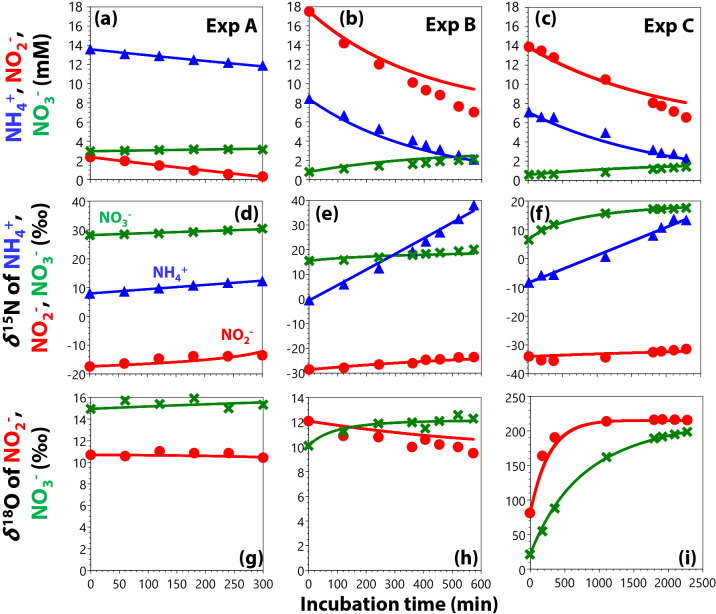
Concentrations and isotopic signatures of inorganic N in incubation experiments. The lines represent changes in the concentrations and isotopic signatures estimated by the curve-fitting of rate constants (for concentrations, upper panels) and ^15^N and ^18^O fractionation factors (for *δ*^15^N and *δ*^18^O values, middle and lower panels). The root mean square error (RMSE) for each fitting was shown in Table S1.

In all experiments, [NH_4_^+^] and [NO_2_^–^] concurrently decreased as [NO_3_^–^] increased (Fig. 2a, b, and c). Averaged stoichiometries during anammox were 1.29, 1.51, and 1.48 for ΔNO_2_^–^ / ΔNH_4_^+^ and 0.16, 0.17, and 0.21 for ΔNO_3_^–^ / ΔNH_4_^+^ in Experiments A, B, and C, respectively (Fig. 2a, b, and c). These results were more consistent in their stoichiometry than previous findings with the same anammox bacterium (1.00 to 2.12 for ΔNO_2_^–^ / ΔNH_4_^+^ and 0.10 to 0.37 for ΔNO_3_^–^ / ΔNH_4_^+^; Ali *et al.*, 2015). The estimated values of *x*, *k*_AMO14N_, and *k*_exch_ were shown in Table S1. The estimated *x* values (0.13 to 0.21; Table S1) were similar to reported values (0.15 to 0.48; Brunner *et al.*, 2013), and *k*_exch_ values were negligible (Table S1). The anammox rates based on NH_4_^+^ consumption were 39.7, 61.7, and 12.4‍ ‍μM (g-S.S.)‍ ‍^–1^‍ ‍h^–1^ for Experiments A, B and C, respectively.

*δ*^15^N_NH4+_, *δ*^15^N_NO2–_, and *δ*^15^N_NO3–_ increased as [NH_4_^+^] and [NO_2_^–^] decreased during anammox (Fig. 2d, e, and f). In contrast, *δ*^18^O_NO2–_ and *δ*^18^O_NO3–_ did not change in Experiment A (Fig. 2g), while *δ*^18^O_NO3–_ increased by ~2‰ and *δ*^18^O_NO2–_ decreased by ~3‰ in Experiment B (Fig. 2h). In Experiment C with the high *δ*^18^O of H_2_O (229‰), *δ*^18^O_NO2–_ and *δ*^18^O_NO3–_ rapidly increased (Fig. 2i). *δ*^18^O_NO2–_ also rapidly increased in the negative control experiment without the biomass (Fig. S3). The isotope exchange between NO_2_^–^ and NO_3_^–^ needs to be taken into consideration (Brunner *et al.*, 2013) when the rapid and large changes in *δ*^15^N and *δ*^18^O at the beginning of the incubation are observed. Since we did not observe such a marked change in *δ*^15^N and *δ*^18^O, indicating isotope exchange (Fig. 2), we did not include isotope exchange between NO_2_^–^ and NO_3_^–^ in the present study.

^15^ε_AMX_ values were calculated as 32.5, 25.4, and 19.3‰ for Experiments A, B, and C, respectively (Table 2, Fig. 2d, e, and f). These ^15^ε_AMX_ values were similar to previously reported values (23.5 ~ 29.1‰) for *Kuenenia stuttgartiensis* in batch incubation experiments (Brunner *et al.*, 2013). ^15^ε_AMXNIR_ values were estimated to be 13.7, 21.8, and 15.6‰ (Table 2, Fig. 2d, e, and f), while ^18^ε_AMXNIR_ values were 3.1, 0 and 60.0‰ (Table 2, Fig. 2d, e, and f) for Experiments A, B and C, respectively. Although ^18^ε_AMXNIR_ values in Experiments B and C were not successfully measured (Table 2; see the Methods), estimated ^15^ε_AMXNIR_ values were consistent with the ^15^N values reported for NO_2_^–^ reduction by Cu-NIR coded by the *nirK* gene (22 ± 2 and 2 ± 2‰) (Martin and Casciotti, 2016). The similarity in these values was attributed to the Cu-NIR of “*Candidatus* Jettenia” with the *nirK* gene (Hira *et al.*, 2012; Ali *et al.*, 2015), the dominant microbe in incubation experiments (Fig. S2).

In addition to normal isotopic fractionation, we estimated ^15^N and ^18^O fractionation factors during anaerobic nitrite oxidization to NO_3_^–^ of –77.8‰ for ^15^ε_NXR_ and –20.6‰ for ^18^ε_NXR_ (Table 2, Fig. 2g, h, and i) in Experiment A. Although we also estimated ^15^N and ^18^O fractionation factors of –65.9 and –71.1‰ for ^15^ε_NXR_ and –11.2 and –84.3‰ for ^18^ε_NXR_ for Experiments B and C, respectively, ^18^ε_NXR_ values for these experiments were not precisely measured. The large inverse ^15^ε_NXR_ is consistent with the reported value with *K. stuttgartiensis* (–31.1 ± 3.9‰; Brunner *et al.*, 2013), as well as aerobic nitrite oxidation to NO_3_^–^ by nitrite-oxidizing bacteria (–12.8 ± 1.5‰; Casciotti, 2009). Regarding oxygen, although only ^18^ε_NXR_ in Experiment A was successfully assessed, the estimated ^18^ε_NXR_ value was negative and close to the inverse ^18^O fractionation factors for aerobic nitrite oxidization to NO_3_^–^ by nitrite-oxidizing bacteria (–10 to –‍1‰; Buchwald and Casciotti, 2010).

### Simulation for denitrification and anammox

**Fig. 4.**
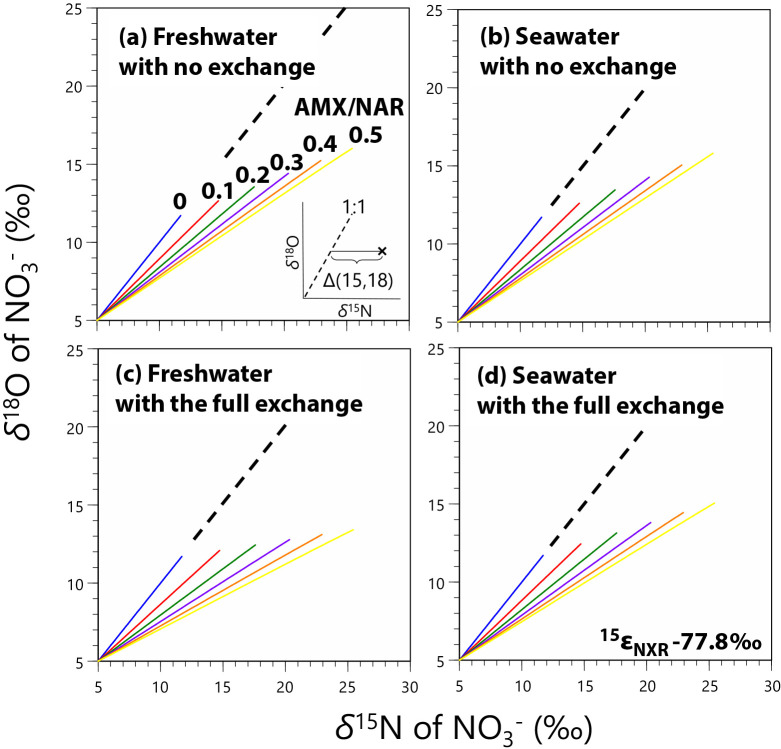
Results from the anammox-denitrification model for variable ratios of anammox (AMX) and denitrification (NAR), and with or without oxygen atom exchange between water and NO_2_^–^. The simulation was run with ^15^ε_NXR_ = –77.8‰ (Table 2) until more than 25% of the initial NO_2_^–^ pool was consumed; however, NO_2_^–^ consumption in simulations with the same run times varied according to the different AMX / NAR ratios. The end point of each simulation run was not important, whereas the slope of each run was. The dotted line in each panel illustrated the denitrification slope (1:1) and the inset in Fig. 4a shows Δ(15, 18) in the *δ*^15^N and *δ*^18^O space.

**Fig. 5.**
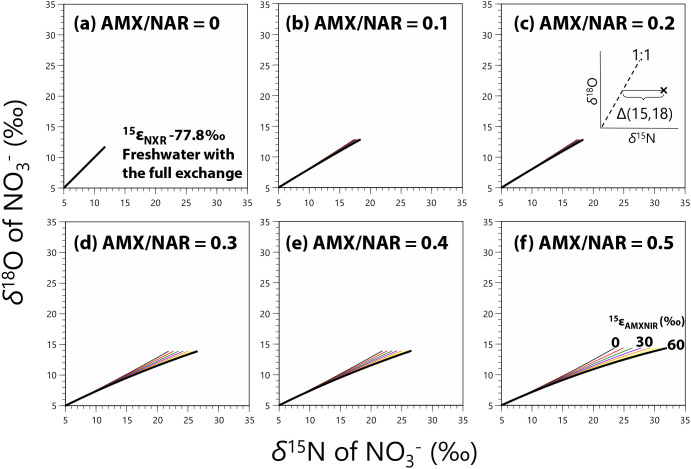
Results of the anammox-denitrification model for variable ANX / NAR ratios with variable ^15^ε_AMXNIR_ values with the full oxygen atom exchange between water (freshwater with *δ*^18^O_H2O_ = –8‰) and NO_2_^–^. The simulation was run with ^15^ε_NXR_ = –77.8‰ (Table 2) until more than 25% of the initial NO_2_^–^ pool was consumed; however, NO_2_^–^ consumption in simulations with the same run times varied according to the different AMX / NAR ratios. The end point of each simulation run was not important, whereas the slope of each run was crucial. The inset in Fig. 5c shows Δ(15, 18) in the *δ*^15^N and *δ*^18^O space.

We developed an anammox-denitrification model with the estimated isotopic fractionation factors (from Experiment A; Table 2) and reported values (Fig. 3) to clarify whether anammox induces a deviation in *δ*^18^O_NO3–_ vs *δ*^15^N_NO3–_ from the denitrification slope of 1 (*i.e.*, Δ(15, 18); defined as (*δ*^15^N – *δ*^15^N_initial_) – (^18^ε / ^15^ε)(*δ*^18^O – *δ*^18^O_initial_), where ^18^ε / ^15^ε is the ratio of isotopic fractionation for O and N during denitrification, respectively, and assigned as 1; see the inset in Fig. 4a; Sigman *et al.*, 2005). Each simulation was run until more than 25% of NO_2_^–^ was consumed. In the case of denitrification in which AMX / NAR is equal to 0 (indicating no anammox), *δ*^18^O_NO3–_ vs *δ*^15^N_NO3–_ was set to show a slope of 1 (the dotted lines in Fig. 4a, d, c, and d). As reported in previous studies (Casciotti and Buchwald, 2012; Granger and Wankel, 2016; He and Bao, 2019), larger anammox rates (larger AMX / NAR) induced greater offsets (larger Δ[15, 18]) from the 1:1 relationship between *δ*^18^O_NO3–_ and *δ*^15^N_NO3–_ in all cases (Fig. 4a, b, c, and d). The effect of oxygen atom exchange was small, but obvious (Fig. 4c for freshwater and Fig. 4d for seawater) with a larger offset with the exchange. Although the present results revealed that Δ(15, 18) is a sensitive parameter for the occurrence of anammox, its usefulness diminishes with smaller ^15^ε_NXR_ values (–31.1‰, Table 2 and Fig. S4), indicating the sensitivity of Δ(15, 18) against ^15^ε_NXR_ (*i.e.*, the stronger ^15^ε_NXR_, the larger Δ [15, 18]). To elucidate the relationship between Δ(15, 18), AMX / NAR, and isotopic fractionation factors, we simulated the *δ*^18^O_NO3–_ and *δ*^15^N_NO3–_ trajectories along with the different AMX / NAR ratios and ^15^ε_AMXNXR_ (Fig. 5 with ^15^ε_NXR_ = –77.8‰ and Fig. S5 with –31.1‰). Similar to ^15^ε_NXR_, stronger ^15^ε_AMXNXR_ resulted in larger Δ(15, 18); however, Δ(15, 18) depended on AMX / NAR, ^15^ε_AMXNXR_, and ^15^ε_NXR_ (Fig. 5 and S5). Therefore, a simple comparison of Δ(15, 18) data does not permit quantitative estimations of AMX / NAR because Δ(15, 18) increases with NO_2_^–^ and NO_3_^–^ consumption whenever anammox is active (AMX / NAR > 0; Fig. 4 and 5) and Δ(15, 18) levels strongly depend on many parameters, including ^15^ε_AMXNXR_ and ^15^ε_NXR_. Although more information on isotopic fractionation factors is needed for quantitative interpretations due to the sensitivity of Δ(15, 18), our simulation exercise revealed that Δ(15, 18), the offset from the 1:1 relationship between *δ*^15^N and *δ*^18^O, may be useful for detecting NXR (nitrite oxidation) in denitrifying systems in both freshwater and seawater.

Besides NXR, some chemolithoautotrophic (*e.g.*, sulfide-dependent) denitrification with auxiliary *Nap* NO_3_^–^ reductase may exhibit a 2:1 rather than 1:1 relationship between *δ*^15^N and *δ*^18^O, resulting in an offset from the 1:1 relationship (Frey *et al.*, 2014). Although this non-respiratory pathway (*i.e.*, *Nap* NO_3_^–^ reduction) is not considered to be a major environmental sink for NO_3_^–^ (Granger and Wankel, 2016), and, thus, was not included in our models, it is worthwhile considering this autotrophic denitrification as a driver of the offset in a sulfide-rich environment in which anammox may be inhibited (Jensen *et al.*, 2008) and sulfide-dependent denitrification enhanced.

## Conclusion

We estimated ^15^N and ^18^O fractionation factors during anammox. The inverse ^15^N effects for NXR (and possibly inverse O isotope effects) may induce an offset from the denitrification trajectory (1:1 relationship between *δ*^15^N and *δ*^18^O of NO_3_^–^, Δ[15, 18]). In practice, Δ(15, 18) may be evaluated with time-course samplings or short incubation studies to investigate the occurrence of anammox, similar to denitrification. This technique will be advantageous because of its potential in evaluations of the quantitative contribution *in situ* of anammox versus denitrification. Although the detection and quantification of functional genes in denitrification and anammox may be readily performed, difficulties are associated with detecting the* in situ* occurrence of denitrification and anammox. Although the isotopic fractionation factors used also need to be considered, Δ(15, 18) is a promising parameter to complement molecular data and the results from laboratory incubation experiments in the study of anammox.

## Citation

Kotajima, S., Koba, K., Ikeda, D., Terada, A., Isaka, K., Nishina, K., et al. (2020) Nitrogen and Oxygen Isotope Signatures of Nitrogen Compounds during Anammox in the Laboratory and a Wastewater Treatment Plant. *Microbes Environ ***35**: ME20031.

https://doi.org/10.1264/jsme2.ME20031

## Supplementary Material

Supplementary Material

## Figures and Tables

**Table 1.** Average concentrations and isotopic compositions of DIN in the anammox plant and isotopic data from different types of WWTP

Reactor	[NH_4_^+^] (mM)	[NO_2_^–^] (mM)	[NO_3_^–^] (mM)	δ^15^N_NH4+_ (‰)	δ^15^N_NO2–_ (‰)	δ^18^O_NO2–_ (‰)	δ^15^N_NO3–_ (‰)	δ^18^O_NO3–_ (‰)	
Influent	44.5 (0.1)	0 (0)	0 (0)	–10.4 (0.2)	n.d.	n.d.	n.d.	n.d.	
DN	40.7 (0.2)	0.2 (0)	0 (0)	–8.4 (0.2)	–27.3 (0.4)	4.7 (0.2)	n.d.	n.d.	
BD	32.8 (0.7)	7.7 (0.6)	0 (0)	0.6 (0.9)	–38.8 (0.8)	4.3 (0.2)	n.d.	n.d.	
NT	18.7 (0.4)	21.2 (0.3)	0 (0)	19.2 (0.7)	–28.2 (0.4)	4.5 (0.1)	n.d.	n.d.	
ANX	1.9 (0.5)	0.4 (0)	2.5 (0)	50.2 (1.4)	–21.6 (0.4)	3.3 (0.3)	9.3 (0.4)	2.7 (0.2)	
WWTP type^#^									Reference
CAS				0 to 36			13 to 15	–1 to 0	Tumendelger *et al.*, 2014
A_2_O							8.1*	–4.5*	Toyoda *et al.*, 2011
Preliminary							11.5 (3.1)**	4.9 (4.2)**	Archana *et al.*, 2016
Primary							14.8 (3.9)**	8.6 (3.4)**	Archana *et al.*, 2016
CEPT							10.6 (4.9)**	–2.1 (3.6)**	Archana *et al.*, 2016
Secondary							12.5 (4.3)**	3.8 (2.4)**	Archana *et al.*, 2016
Tertiary							90.7 (83.9)**	87.7 (90.6)**	Archana *et al.*, 2016

Means from three sampling times with standard errors (in parentheses) are shown. n.d.: not determined * Data from the sampling point closest to the outlet to the river ** Means from several WWTP with standard deviations (in parentheses) are shown. ^#^ CAS: Conventional activated sludge, A_2_O: Anaerobic-Anoxic-Oxic treatment, CEPT: Chemically Enhanced Primary Treatment

**Table 2.** Isotopic fractionation factors during anammox (‰)

Open system	Anammox Plant (This study)				Reported values
	20150428	20150507	20150512		Kobayashi *et al.*, (2019)
^15^Δ_AMXNIR_	11.8	12.0	12.4		5.9~29.5
^15^Δ_NXR_	–30.4	–31.1	–31.3		–30.1~ –45.3
^15^Δ_AMX_	34.0	34.8	34.4		30.9~32.7
^18^E_AMXcombined_*	–3.8	–2.5	–3.2		–1.5~ –12.1
Closed system	Batch incubations (This study)				Reported values
	Experiment A	Experiment B	Experiment C		Brunner *et al.*, (2013)
^15^ε_AMXNIR_	13.7	21.8	15.6		16.0
^15^ε_NXR_	–77.8	–65.9	–71.1		–31.1
^15^ε_AMX_	32.5	25.4	19.3		23.5~29.1
^18^E_AMXcombined_*	–10.4	n.d.	n.d.		n.d.
^18^ε_AMXNIR_**	3.1	n.d.	n.d.		n.d.
^18^ε_NXR_**	–20.6	n.d.	n.d.		n.d.

*: ^18^ε_NXR_ × 2 / 3 + ^18^ε_H2ONXR_ / 3 (Kobayashi *et al.*, 2019) **: assuming ^18^ε_EQ_ = 1.013, ^18^ε_H2ONXR_ = 1.010 (Table S2)
