# Supplementary material for: Nitrogen and Oxygen Isotope Signatures of Nitrogen Compounds during Anammox in the Laboratory and a Wastewater Treatment Plant
Source: Microbes Environ. 2020 Nov 7;35(4):ME20031. doi: 10.1264/jsme2.ME20031 (PMC7734408; doi:10.1264/jsme2.ME20031)
Supplement: Supplementary file 1 — Supplementary Material [file 35_20031_s1.pdf]

# Nitrogen and oxygen isotope signatures of nitrogen compounds during anammox in the lab and in a wastewater treatment plant

**S. Kotajima[1], K. Koba[2,3]*, D. Ikeda[4], A. Terada[5, 6], K. Isaka[7,8], K. Nishina[9], Y. Kimura[7], A. Makabe[3,10,**], M. Yano[1,2], H. Fujitani[11,***], N. Ushiki[11], S. Tsuneda[11], M. Yoh[3].**

**Affiliations**

1. Graduate School of Agriculture, Tokyo University of Agriculture and Technology, Tokyo, 1838509, Japan
2. Center for Ecological Research, Kyoto University, Shiga, 5202113, Japan
3. Institute of Agriculture, Tokyo University of Agriculture and Technology, Tokyo, 1838509, Japan
4. Graduate School of Engineering, Tokyo University of Agriculture and Technology, Tokyo, 1848588, Japan
5. Department of Chemical Engineering, Tokyo University of Agriculture and Technology, Tokyo, 1848588, Japan
6. Institute of Global Innovation Research, Tokyo University of Agriculture and Technology, Tokyo, 1858538, Japan
7. Hitachi, Ltd., Chiba, 2710064, Japan
8. Department of Applied Chemistry, Faculty of Science and Engineering, Toyo University, Saitama, 3508585, Japan
9. Center for Regional Environmental Research, National Institute of Environmental Sciences, Ibaraki, 3058506, Japan
10. Project Team for Development of New-generation Research Protocol for Submarine Resources, Japan Agency for Marine-Earth Science and Technology, Kanagawa, 2370061, Japan
11. Department of Life Science and Medical Bioscience, Waseda University, Tokyo, 1628480, Japan

** Present address

Institute for Extra-cutting-edge Science and Technology Avant-garde Research (X-star)

Super-cutting-edge Grand and Advanced Research (SUGAR) Program, Japan Agency for Marine Earth Science and Technology, Kanagawa, 2370061, Japan

*** Present address

Department of Biological Sciences,Faculty of Science and Engineering,Chuo University, Tokyo, 112-8551, Japan

## SI Text

### 1.1. The details on a small-scale anammox reactor including the start-up information, maintenance, performance, input solutions and the microbial communities of the reactor.

The biomass samples highly enriched with anammox bacteria were taken from an upflow-column bed reactor where non-woven sheet was mounted as a carrier material (Isaka *et al.*, 2007). The reactor with an effective volume of 50 L continuously received a synthetic medium with an ammonium and nitrite concentrations of each 250 mg-N/L. The medium consisted of 250 mg-N/L of $(NH_4)_2SO_4$, 250 mg-N/L of $NaNO_2$, 540 mg/L of $NaHCO_3$, 32.4 mg/L of $KH_2PO_4$ 32.4 mg/L, 36 mg/L of $MgSO_4 \cdot 7H_2O$, 216 mg/L of $CaCl_2 \cdot 2H_2O$, and 1 mL/L of trace element solution with the compositions as previously reported (de Graaf *et al.*, 1996). A hydraulic retention time, temperature, and pH were kept at 0.5 day, 30-35°C, and 7.5-8.0, respectively.

For the analysis of microbial community composition, the biomass sample from the reactor was taken, subject to DNA extraction by FastDNA spin kit for Soil (MP Bio, CA) according to the manufacturer's protocol. The extracted DNA was applied to PCR using primer 1055f-1392r for V7-V8 hypervariable region of 16S the rRNA gene. Amplicon sequencing was performed using an Ion Personal Genome Machine Sequencer (Ion Torrent; Thermo Fisher Scientific, Waltham, MA). The subsequent processing of the attained sequences was conducted by QIIME (Caporaso *et al*., 2010). The operational taxonomic unit (OTU) was defined by a 97% sequence similarity. The detailed protocol was after the previous work (Abe *et al.*, 2017). The amplicon sequencing data of the 16S rRNA data was registered on DDBJ Sequence Read Archive (Bioproject: PRJDB9482).

The microbial community compositions of the biomasses are summarized in Figure S2. The predominant anammox bacteria were consistently affiliated with the genus *Candidatus* Jettenia, accounting up to 54.8% and 80.8% in the biomasses for the batch tests of A and C, respectively. These biomasses also harbored heterotrophic bacteria. Betaproteobacteria was the most abundant among classes for heterotrophic bacteria. The relative abundances of the family Rhodocyclaceae, reported canonical denitrifying bacteria (Ginige *et al.*, 2005), were 9.7% and 5.5% for the batch tests A and C, respectively.

Reference cited in this section

Abe, T., Ushiki, N., Fujitani, H., and Tsuneda, S. (2017) A rapid collection of yet unknown ammonia oxidizers in pure culture from activated sludge. *Water Res* **108**, 169-178.

Caporaso, J.G., Kuczynski, J., Stombaugh, J., Bittinger, K., Bushman, F.D., Costello, E.K., *et al.* (2010) QIIME allows analysis of high-throughput community sequencing data. *Nature Methods* **7**(5), 335-336.

Ginige, M.P., Keller, J., and Blackall, L.L. (2005) Investigation of an acetate-fed denitrifying microbial community by stable isotope probing, full-cycle rRNA analysis, and fluorescent in situ hybridization-microautoradiography. *Appl Environ Microbiol* **71**(12), 8683-8691.

Isaka, K., Sumino, T., and Tsuneda, S. (2007) High nitrogen removal performance at moderately low temperature utilizing anaerobic ammonium oxidation reactions. *J Biosci Bioeng* **103**(5), 486-490.

van de Graaf, A.A.V., deBruijn, P., Robertson, L.A., Jetten, M.S.M., and Kuenen, J.G. (1996) Autotrophic growth of anaerobic ammonium-oxidizing micro-organisms in a fluidized bed reactor. *Microbiology (Reading, England)* **142**, 2187.

SI Figures

Fig. S1

Block flow diagram of Full-scale Anammox plant (after Isaka *et al*., 2017) and the sampling points

Fig. S2

Microbial community of the biomass used in the incubation experiments

Fig. S3

Concentrations and isotopic signatures of inorganic N in the incubation experiments without the activated sludge as the negative control

Fig. S4

Results of anammox-denitrification model for variable ratios of anammox (AMX) and denitrification (NAR), and with or without oxygen atom exchange between water and $NO_2^-$ with $^{15}\varepsilon_{NXR}$ = -31.1 ‰ (after Brunner *et al*., 2013). The simulation was run until more than 25% of the initial $NO_2^-$ pool was consumed, but $NO_2^-$ consumption in the simulations with the same run times varied according to the different AMX/NAR ratios. The end point of each simulation run is not important but the slope of each run is important. The dotted line in each panel illustrated the denitrification slope (1:1) and the inset in Fig. 4a illustrate Δ(15,18) in $\delta^{15}$N and $\delta^{18}$O space.

Fig. S5

Results of anammox-denitrification model for variable ANX/NAR ratios with variable $^{15}\varepsilon_{AMXNIR}$ values with the full oxygen atom exchange between water (freshwater with $\delta^{18}O_{H2O}$ = -8 ‰) and $NO_2^-$. The simulation was run with $^{15}\varepsilon_{NXR}$ = -31.1 ‰ (after Brunner *et al*., 2013) until more than 25% of the initial $NO_2^-$ pool was consumed, but $NO_2^-$ consumption in the simulations with the same run times varied according to the different AMX/NAR ratios. The end point of each simulation run is not important but the slope of each run is important. The inset in Fig. S5c illustrates Δ(15,18) in $\delta^{15}$N and $\delta^{18}$O space.

Table S1

Parameters used in the simulations in this study

Table S2

RMSE values for each parameter in the incubation experiments

Fig. S1 Block flow diagram of Full-scale Anammox plant and the sampling points

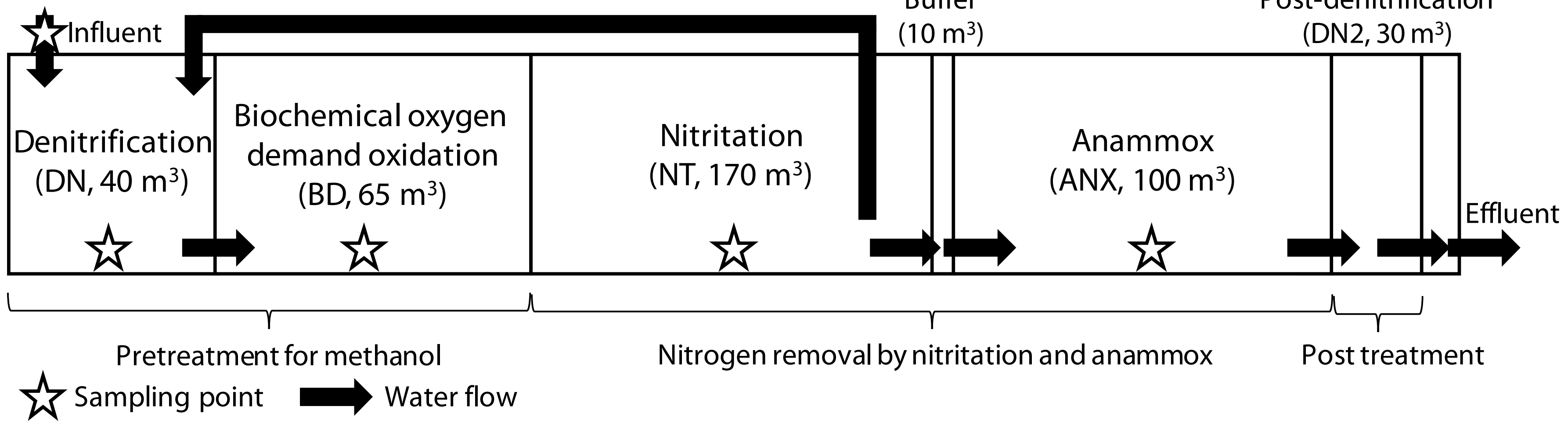


Fig. S1 Block flow diagram of full-scale anammox plant. Denitrification (DN) and biochemical oxygen demand oxidation (BD) reactor were mainly used as pretreatment process for removing the strong anammox inhibitor (methanol). About half of ammonium was oxidized to nitrite in the partial nitrification reactor (NT). Subsequently, ammonium and nitrite were both converted into nitrogen gas in the anammox reactor (ANX). To maintain the bacteria in each reactor carrying out denitrification, methanol oxidation, nitrification and anammox, gel carriers which entrapped the bacteria were installed. The flow rate of ammonia wastewater for anammox reactor was maintained at 660 $m^3$ $day^{-1}$ during sampling periods.

Fig. S2

Microbial community of the biofilm used in Experiments A and C (a) and the granule biomass (b) used in Experiment B used in the incubation experiments.

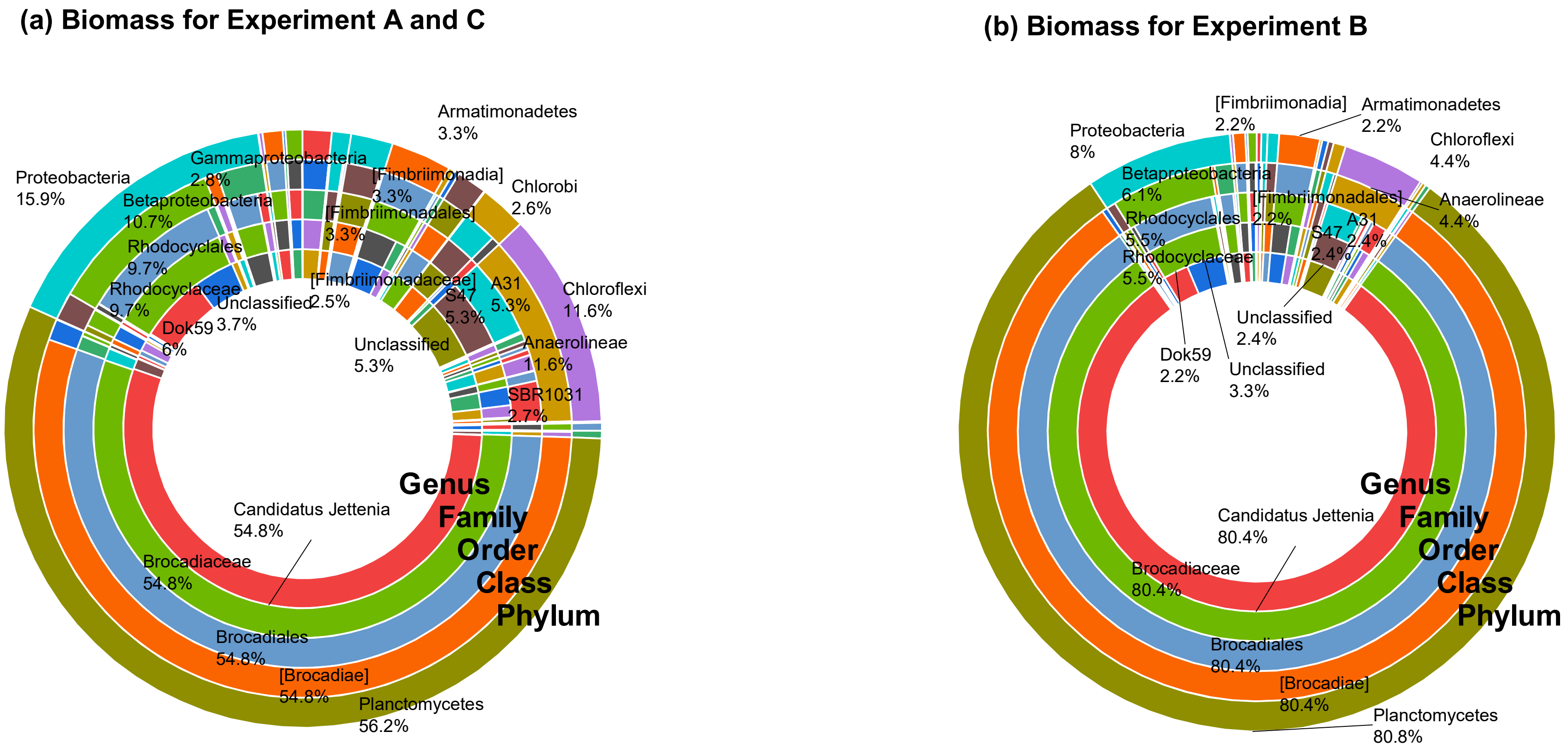

Fig. S3

Concentrations (a), $\delta^{15}N$ (b), and $\delta^{18}O$ (c) of inorganic N in the incubation experiments without the anammox biomass as the negative control. Symbols: ammonium, blue triangles; nitrite, red circles; nitrate, green squares.

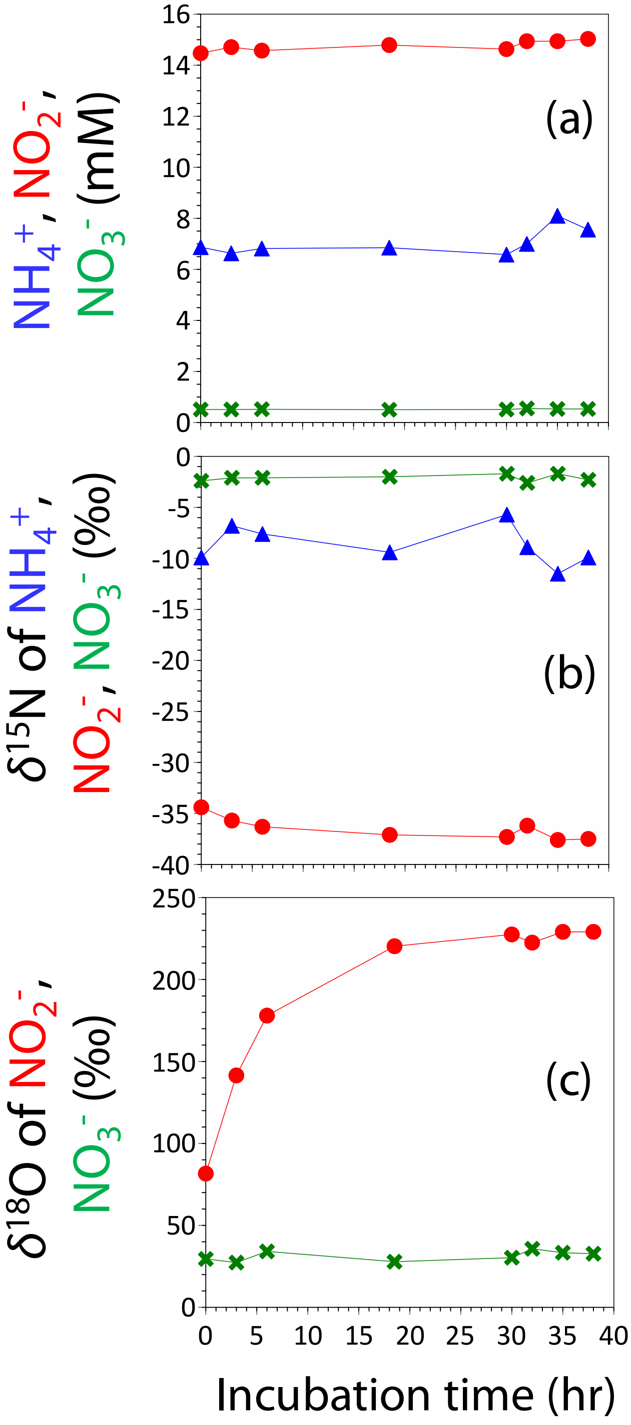

Fig. S4

Results of anammox-denitrification model for variable ratios of anammox (AMX) and denitrification (NAR), and with or without oxygen atom exchange between water and $NO_2^-$ with $^{15}\varepsilon_{NXR}$ = -31.1 ‰ (after Brunner *et al*., 2013) The simulation was run until more than 25% of the initial $NO_2^-$ pool was consumed, but $NO_2^-$ consumption in the simulations with the same run times varied according to the different AMX/NAR ratios. The end point of each simulation run is not important but the slope of each run is important. The dotted line in each panel illustrated the denitrification slope (1:1) and the inset in Fig. S4a illustrate Δ(15,18) in $\delta^{15}$N and $\delta^{18}$O space.

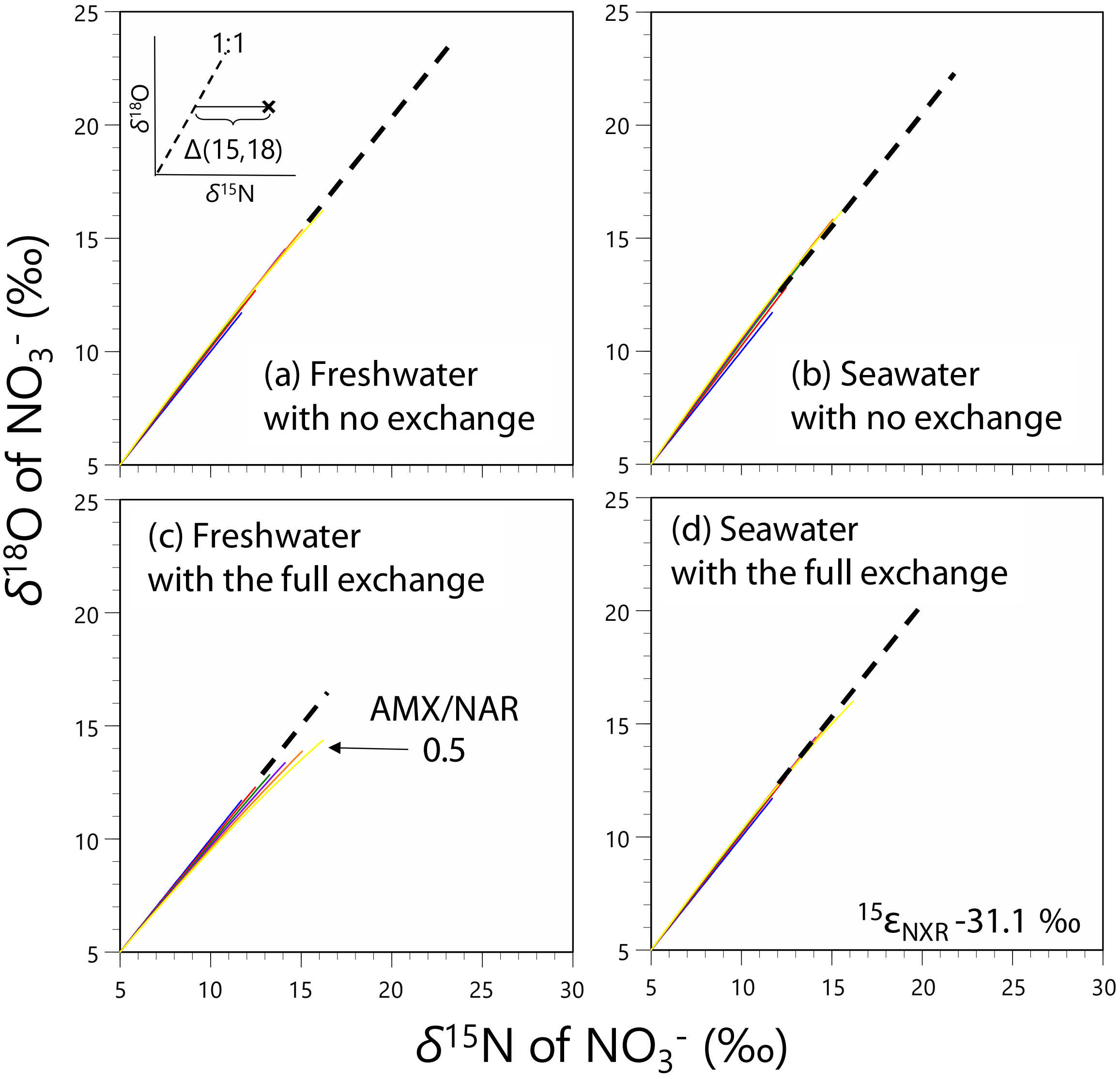

Fig. S5

Results of anammox-denitrification model for variable ratios of anammox (AMX) and denitrification (NAR), and with or without oxygen atom exchange between water and $NO_2^-$ with $^{15}\varepsilon_{NXR}$ = -31.1 ‰ (after Brunner *et al.*, 2013). The simulation was run until more than 25% of the initial $NO_2^-$ pool was consumed, but $NO_2^-$ consumption in the simulations with the same run times varied according to the different AMX/NAR ratios. The end point of each simulation run is not important but the slope of each run is important. The inset in Fig. 4a illustrates Δ(15,18) in $\delta^{15}$N and $\delta^{18}$O space.

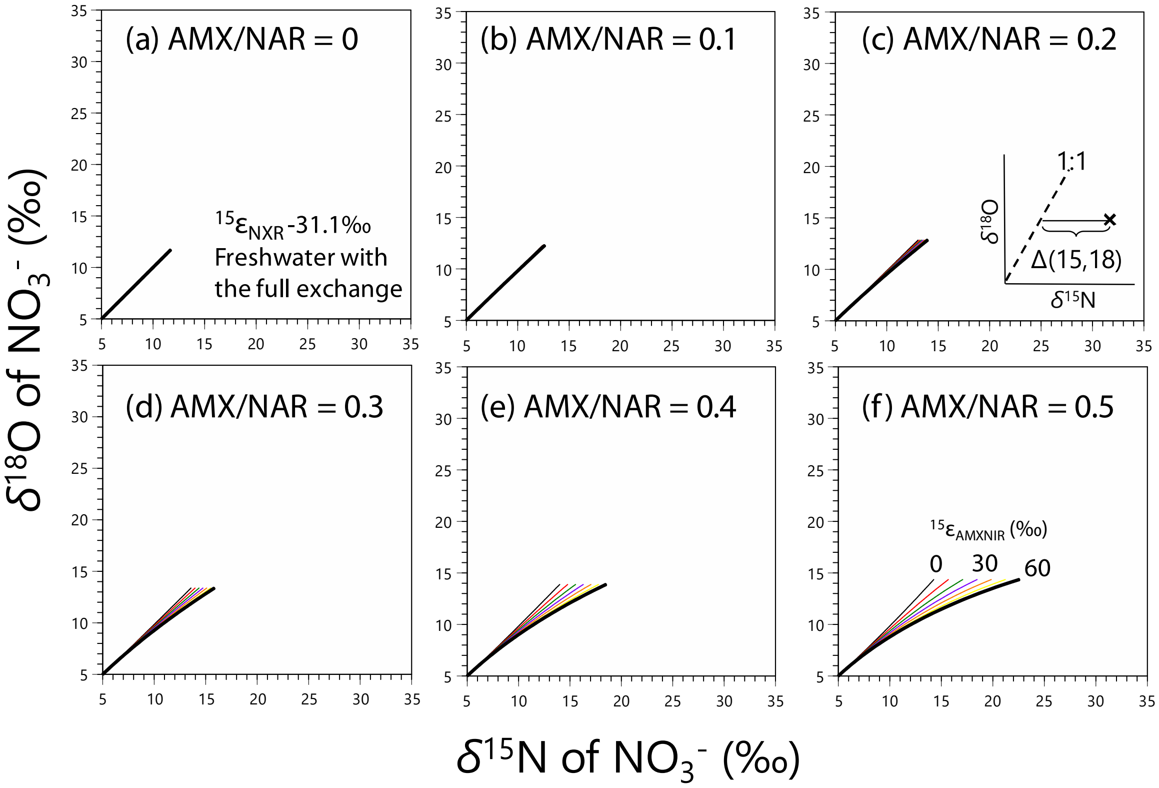

Table S1. Parameters in the anammox model

| Parameters | | Exp. A | Exp. B | Exp. C |
|---|---|---|---|---|
| Initial conditions | | | | |
| | $[NO_2^-]$ (mM) | 2.35 | 17.50 | 7.11 |
| | $[NO_3^-]$ (mM) | 2.96 | 0.79 | 13.90 |
| | $[NH_4^+]$ (mM) | 13.60 | 8.41 | 0.58 |
| | $\delta^{15}N_{NO2-}$ (‰) | -17.4 | -28.6 | -34.0 |
| | $\delta^{15}N_{NO3-}$ (‰) | 28.2 | 15.4 | 6.5 |
| | $\delta^{15}N_{NH4+}$ (‰) | 8.0 | -0.5 | -8.3 |
| | $\delta^{18}O_{NO2-}$ (‰) | 10.7 | 12.1 | 81.9 |
| | $\delta^{18}O_{NO3-}$ (‰) | 14.9 | 10.1 | 22.0 |
| | $\delta^{18}O_{H2O}$ (‰) | -8.0 | -8.0 | 229.0 |
| Assigned | | | | |
| | $^{18}\varepsilon_{EQ}$ (‰) | 13.0 | 13.0 | 13.0 |
| | $^{18}\varepsilon_{H2ONXR}$ (‰) | 10.0 | 10.0 | 10.0 |
| | $^{1518}\varepsilon_{NAR}$ | 15.0 | 15.0 | 15.0 |
| | $^{15}\varepsilon_{DENNIR}$ | 5.0 | 5.0 | 5.0 |
| | $^{18}\varepsilon_{H2OBRNAR}$ | 25.0 | 25.0 | 25.0 |
| | $R_{\_Oxygen}$ | 0.002005 | 0.002005 | 0.002005 |
| | $R_{\_Nitrogen}$ | 0.003677 | 0.003677 | 0.003677 |
| | Temperature (K) | 300 | 300 | 300 |
| Estimated | | | | |
| | $x$ | 0.131 | 0.207 | 0.159 |
| | $k_{AMO14N}$ (/hr) | 4.68E-15 | 2.52E-14 | 5.10E-15 |
| | $k_{exch}$ (/hr) | 2.78E-34 | 1.01E-20 | 3.63E-14 |
| | $^{15}\varepsilon_{AMXNIR}$ (‰) | 13.7 | 21.8 | 15.6 |
| | $^{15}\varepsilon_{NXR}$ (‰) | -77.8 | -65.9 | -71.1 |
| | $^{15}\varepsilon_{AMX}$ (‰) | 32.5 | 25.4 | 19.3 |
| | $^{18}\varepsilon_{AMXNIR}$ (‰) | 3.1 | n.d. | n.d. |
| | $^{18}\varepsilon_{NXR}$ (‰) | -20.6 | n.d. | n.d. |

Table S2. RMSE values for each parameter in the incubation experiments

| Experiment | $[NH_4^+]$ | $[NO_2^-]$ | $[NO_3^-]$ | $\delta^{15}N_{NH4+}$ | $\delta^{15}N_{NO2-}$ | $\delta^{15}N_{NO3-}$ | $\delta^{18}O_{NO2-}$ | $\delta^{18}O_{NO3-}$ |
|---|---|---|---|---|---|---|---|---|
| A | 6.53E-03 | 7.49E-03 | 4.08E-03 | 1.26E-01 | 1.11E+00 | 1.67E-01 | 2.25E-01 | 4.13E-01 |
| B | 4.80E-02 | 1.43E-01 | 3.78E-02 | 1.78E+00 | 5.52E-01 | 8.26E-01 | n.d. | n.d. |
| C | 4.43E-02 | 8.20E-02 | 1.76E-02 | 1.03E+00 | 1.02E+00 | 2.41E-01 | n.d. | n.d. |
